# Supplementary material for: Asymmetric learning and adaptability to changes in relational structure during transitive inference
Source: Commun Psychol. 2025 Nov 14;3:155. doi: 10.1038/s44271-025-00352-0 (PMC12618241; doi:10.1038/s44271-025-00352-0)
Supplement: Supplementary file 2 — Supplementary Material [file 44271_2025_352_MOESM2_ESM.pdf]

Asymmetric learning and adaptability to changes in relational structure during transitive inference – Supplementary Information

Thomas A. Graham, Bernhard Spitzer

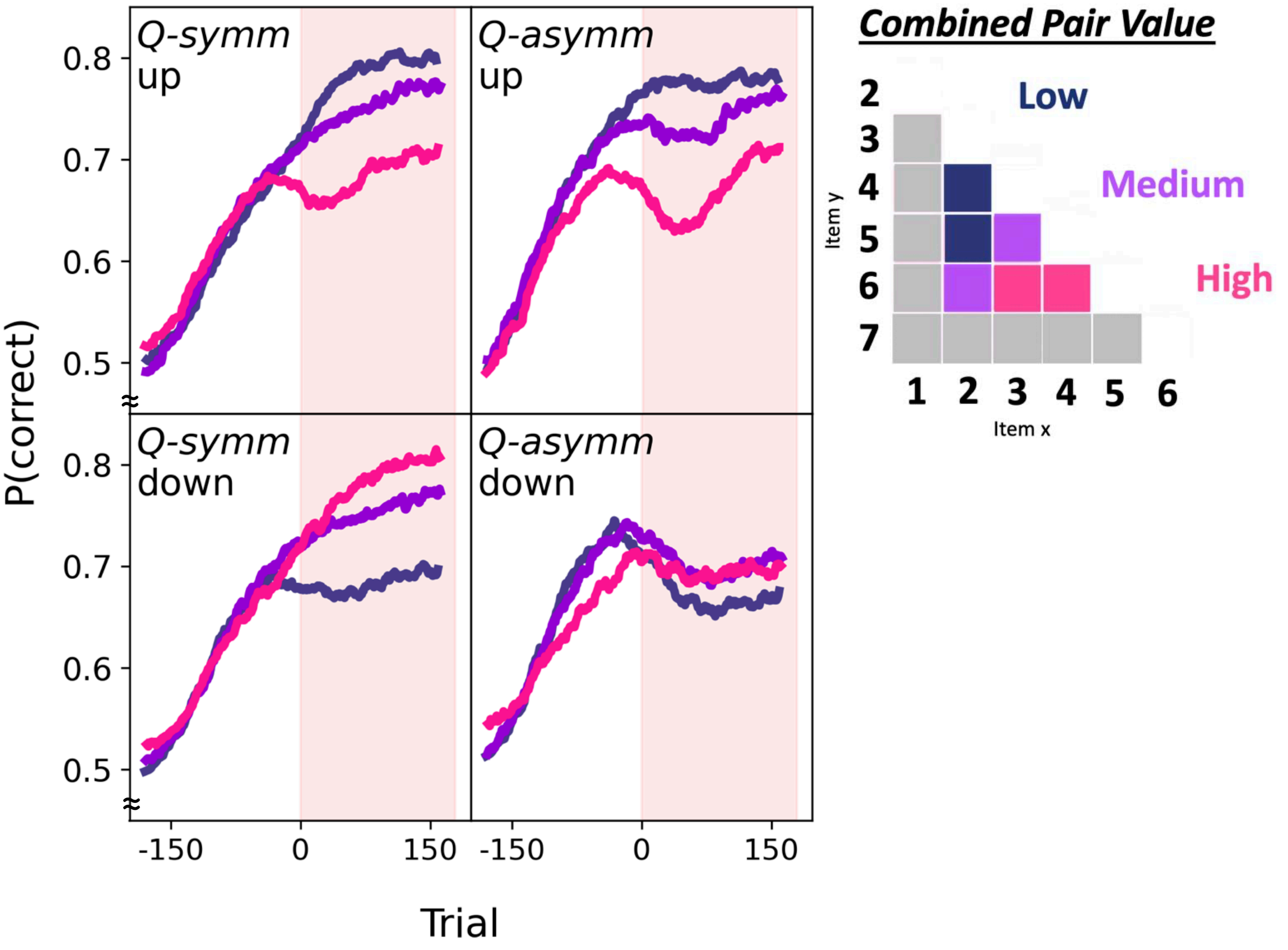

Fig S1. Predicted TI learning curves under simulations of symmetric and asymmetric RL models. Same as Fig 2, but simulated using empirical parameter estimates for *Q-symm* (upper panels) and *Q-asymm* (lower panels) matching those observed by Ciranka et al. [1].

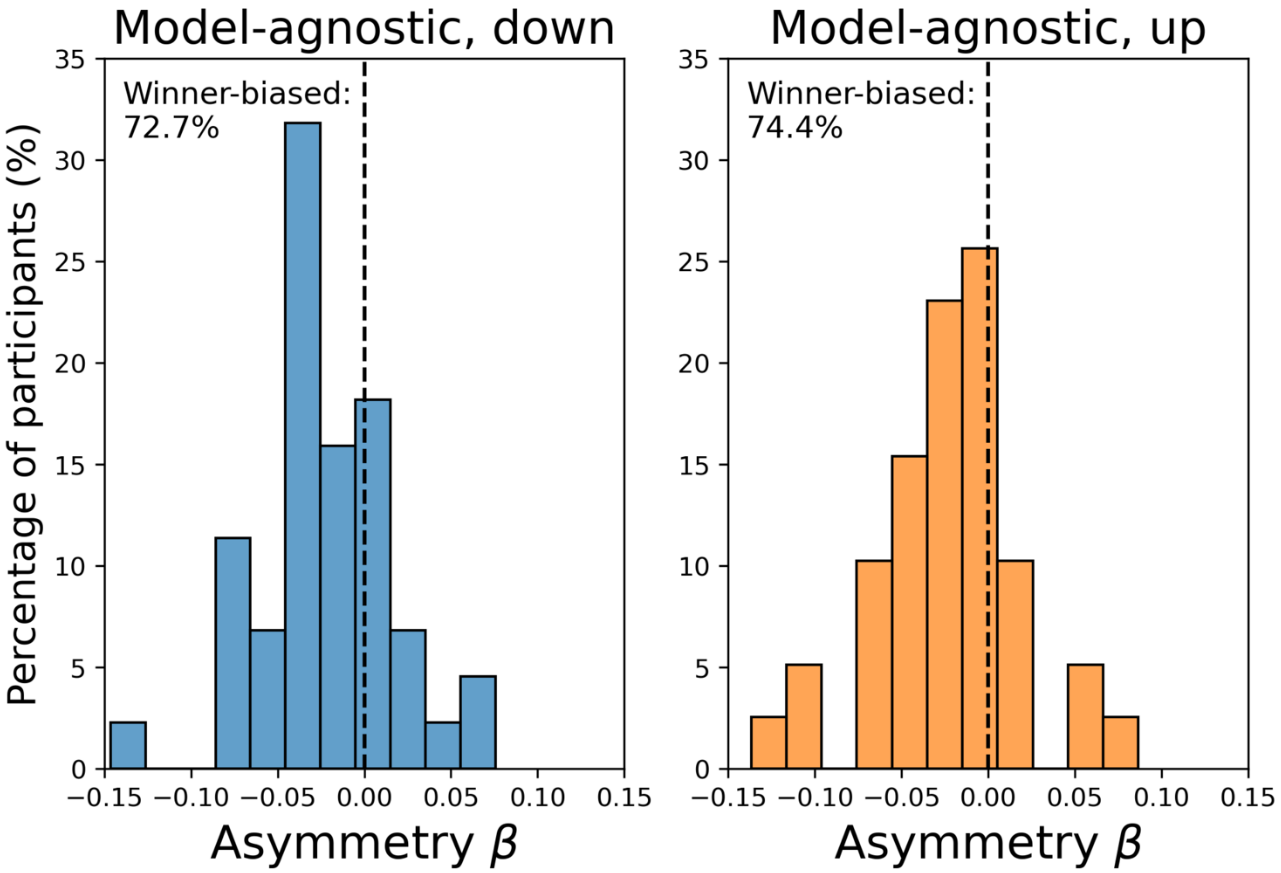

Fig S2. Value compression effects in human behaviour. Our model-agnostic measure of learning asymmetry – i.e. the slope of the relationship between combined item value and accuracy on pre-changepoint TI trials – was significantly lower than 0, indicating value compression. In-text percentages refer to the percentage of participants in each group whose asymmetry slope was below 0, thereby indicating those participants designated as winner-biased (cf. Fig 4A).

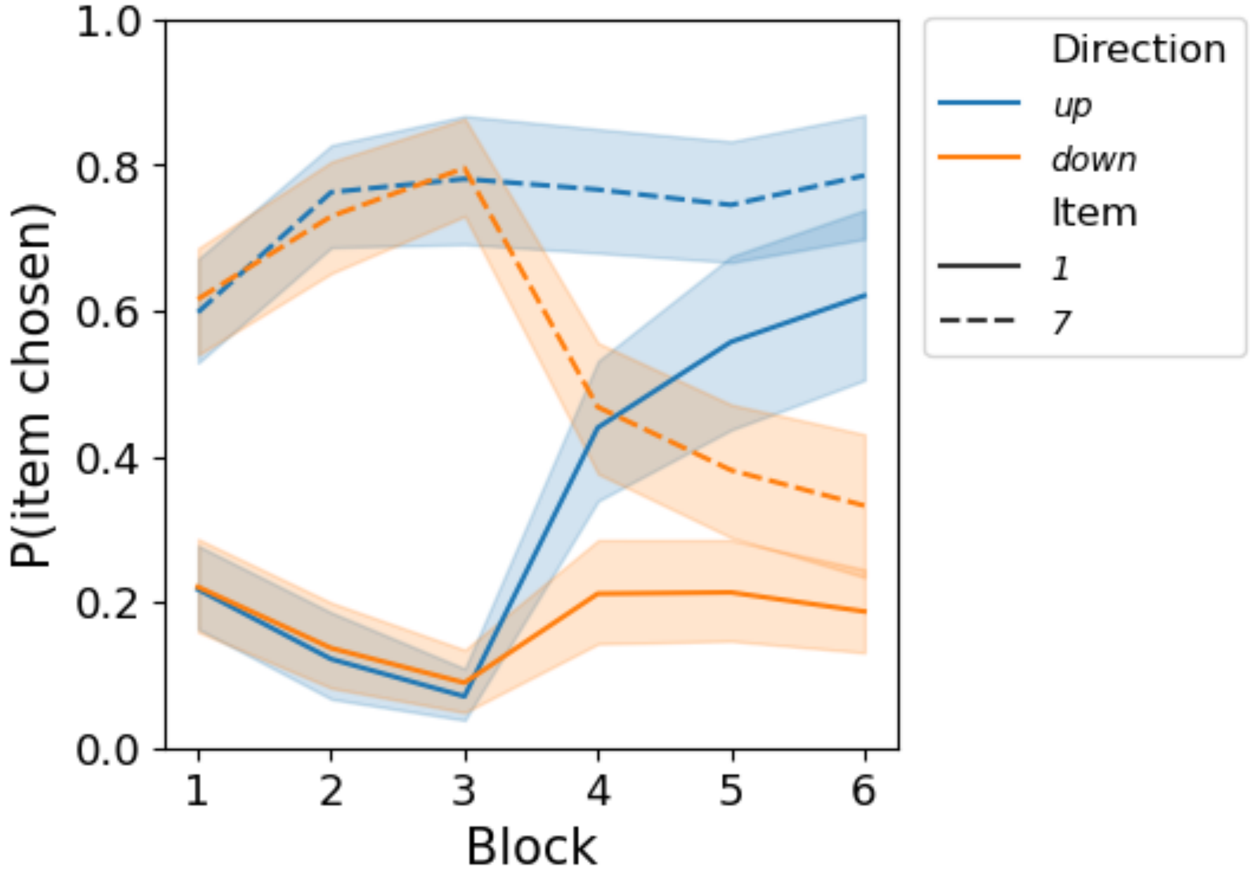

Fig S3. Change in participants' block-wise choice preferences for anchor items. Both sets of participants appropriately adjusted their preferences for the moved anchor item following the changepoint (i.e. increased preference i_1 for 'up' participants, and decreased preference i_7 for 'down' participants). In addition, both groups exhibited differences in how their preferences for the *unmoved* anchor item changed as a result of the changepoint - whereas 'up' participants' preference for i_7 remained stable, 'down' participants increased their preference for i_1. Thus, although the ranks of these unmoved anchors did not change, 'down' participants nonetheless exhibited a bias towards increasing their tendency to choose the unmoved anchor i_7, an effect that 'up' participants did not show for their respective unmoved anchor i_1. Error bars indicate 95% confidence intervals for mean choice probability per block.

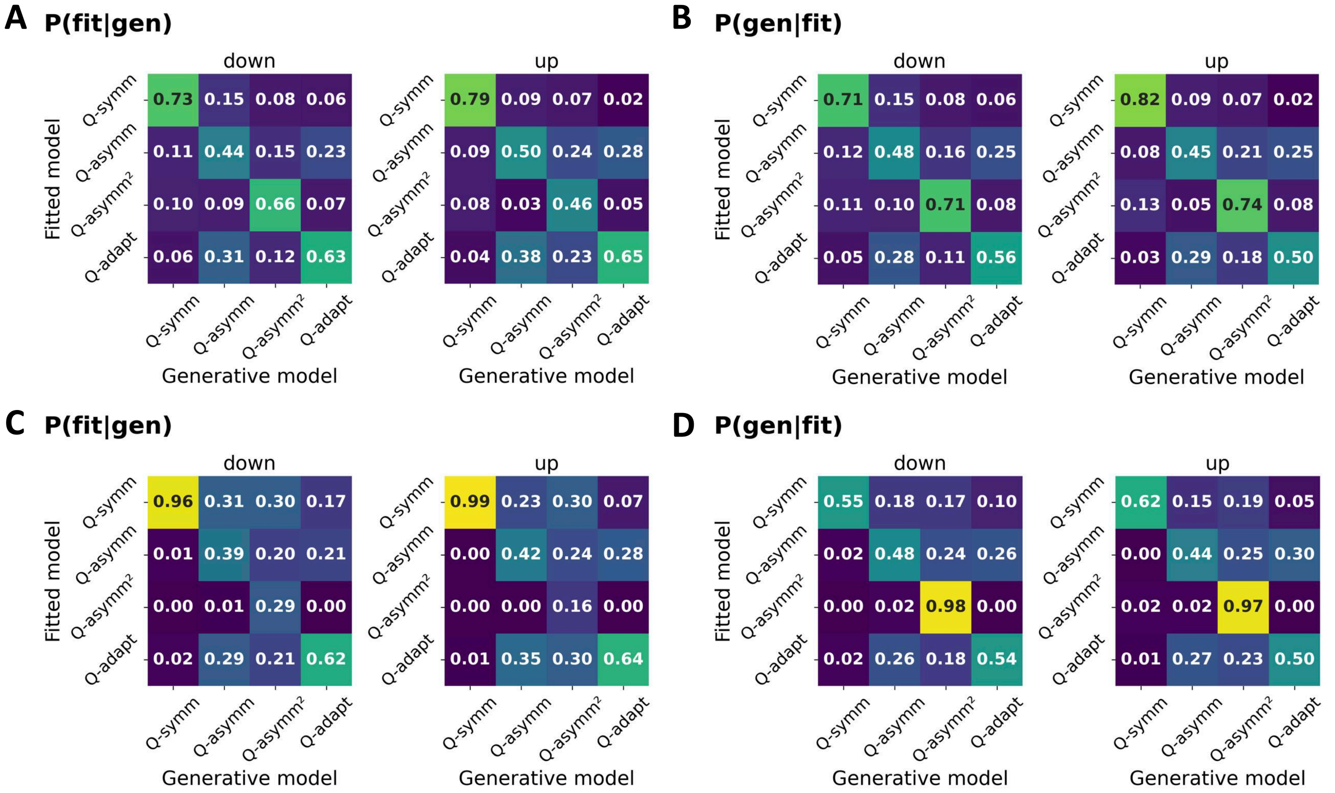

Fig S4A-D. Model recovery analysis. Under AIC, our three candidate models generally exhibited good identifiability both in terms of *p(fit|gen)* (**A**) and *p(gen|fit)* (**B**). In contrast, BIC tended to overpenalise more complex models with more free parameters, leading to more confusion with less complex models (**C** and **D**). See 'Model and Parameter Recovery' in *Supplementary Methods* for details.

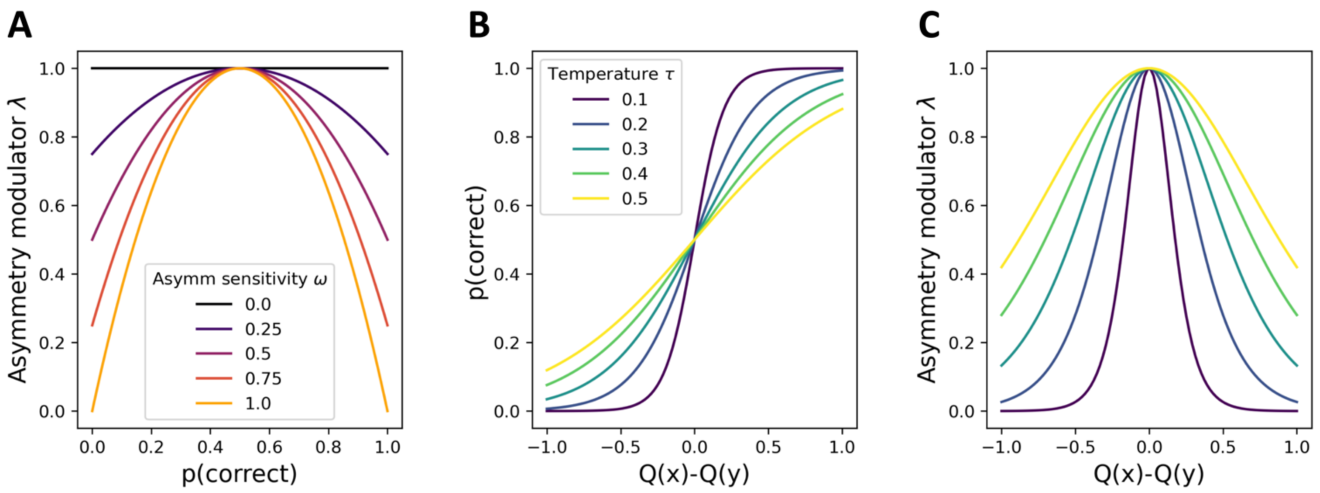

Fig S5A-C. Illustration of how *Q-adapt* modulates its degree of learning asymmetry. A) The asymmetry modulator λ is given by a quadratic function of the agent's preference strength – that is, the probability that they will choose $i_x > i_y$ on a given trial. The steepness of the asymmetry modulator function – that is, the degree to which λ is sensitive to changes in choice probability – is modulated by ω. **B)** Preference strength is a logistic choice function of the difference in value estimates for the compared items, the slope of which is determined by the temperature parameter τ. **C)** Assuming a constant ω (here, ω=1), then, given the relationship between λ and choice preference in **A**, which is itself dependent on τ, this means that the extent to which a difference in value estimates results in a smaller value of λ, and hence a more symmetric learning update, is at least partially shaped by each agent's value for τ, and hence by their decision noise. In practice, *Q-adapt*'s learning dynamics can be roughly described as follows: at the beginning of the experiment, item values are not distinguishable, causing the agent to update items asymmetrically. As learning progresses and stronger preferences are formed, the agent begins to utilise a more symmetric update. Lower noise agents will exhibit a stronger tendency in this direction, meaning that, upon receipt of the $i_7 < i_1$ feedback, they will more appropriately update these items (and indeed items on following trials) in a symmetric fashion, and thus resolve the 'down' changepoint with less difficulty. In contrast, higher noise agents will tend to update more asymmetrically across all value differences, leading to inflexible adaptation to the 'down' changepoint among those who are winner-biased.

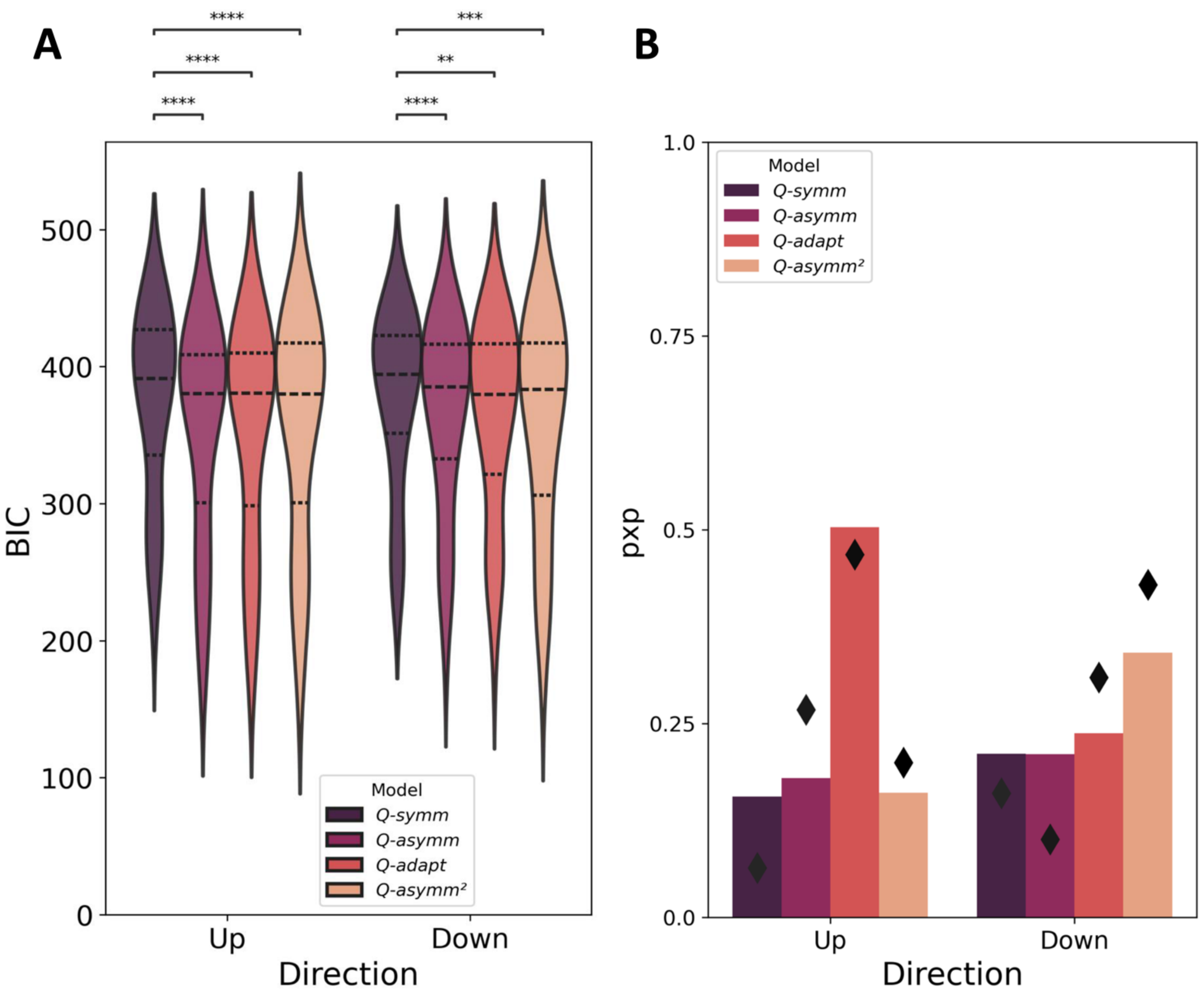

Fig S6A-B. Model comparison for each candidate model, within each task condition, under the alternative model evidence metric BIC. In other words, same as Fig 5A-B, but plotting BIC (**A**) rather than AIC, and then the pxp (bars) and model frequencies (diamonds) derived from these BIC scores (**B**).

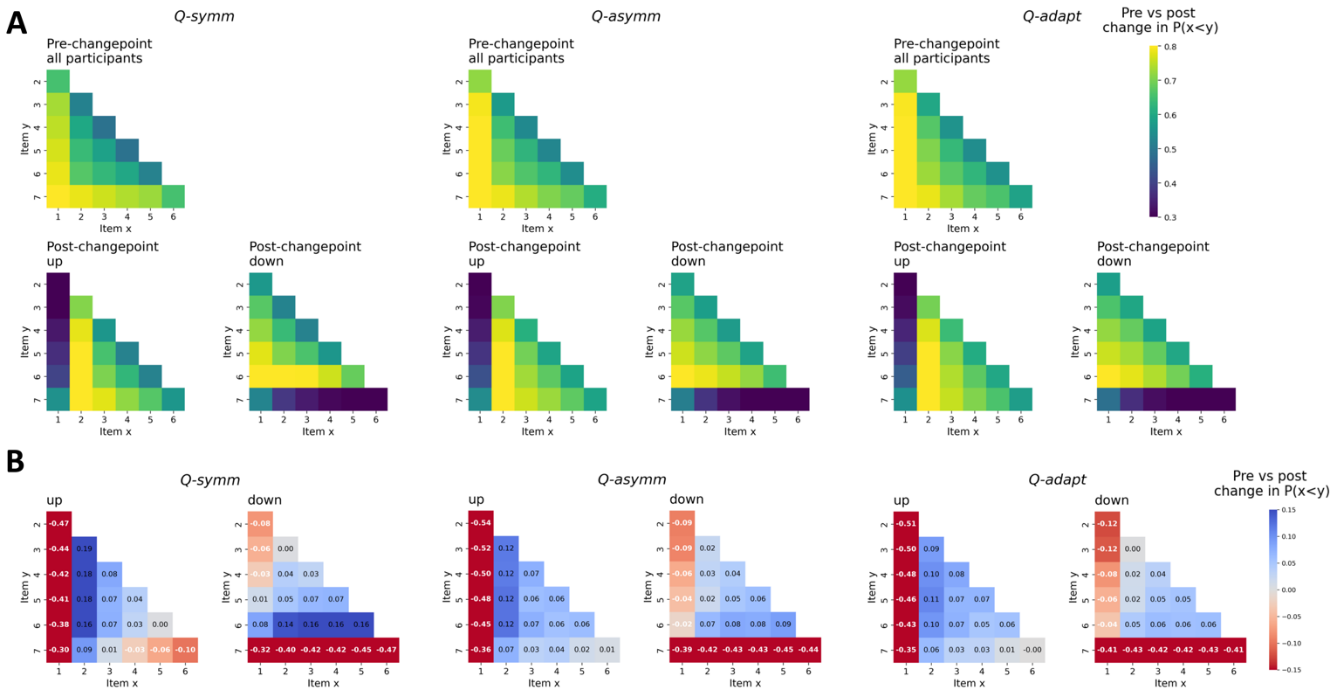

Fig S7A-B. Choice matrices for other candidate models. Same as Fig 3A-B, except for the other three candidate models, i.e. *Q-symm* (left-most panels), *Q-asymm* (central panels) and *Q-adapt* (right-most panels). **A)** Mean probability of choosing item *y* (matrix rows) over item *x* (matrix columns), with the top row of panels displaying pre-changepoint data collapsed across 'up' and 'down' participants, and the bottom row of panels splitting post-changepoint data by group. **B)** Pre vs. post-changepoint change in choice preferences.

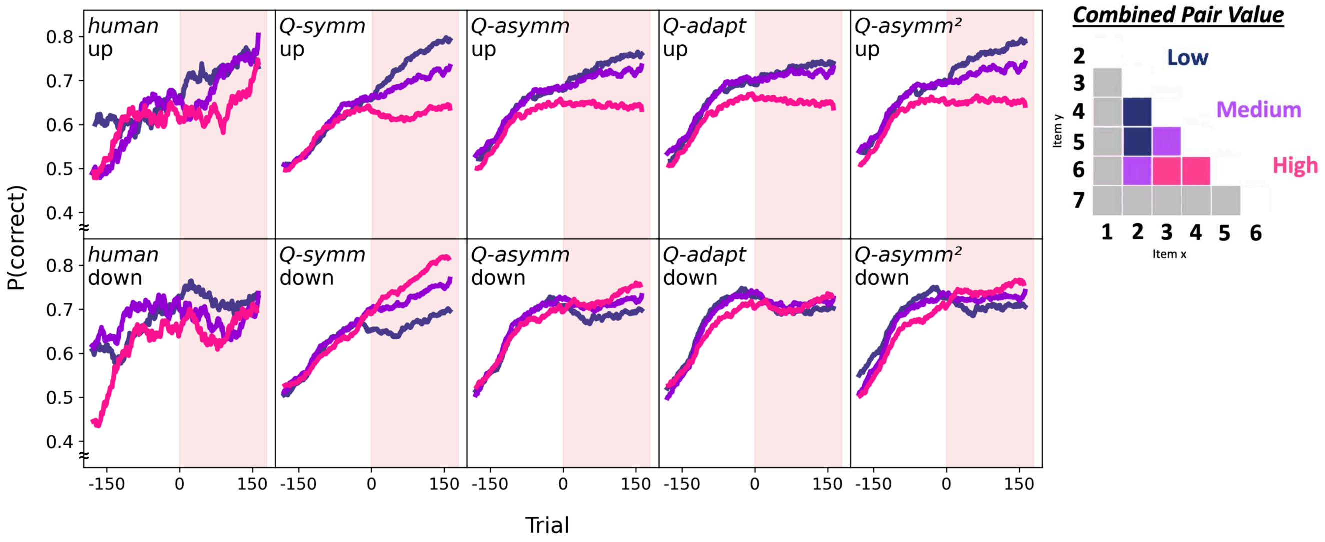

Fig S8. TI accuracy over the course of experiment in humans and fitted models for the larger participant set. Same as Fig 2, but for the participant sample obtained after the application of a more liberal performance-related exclusion criterion (i.e. N = 103, rather than N = 83). As in the more conservative sample reported in the main text, we observed a differential impact of the changepoint on downstream TI performance, as indicated by a significant changepoint x direction interaction effect on non-anchor TI accuracy ($F(1,101) = 5.19$, $p = .025$, $\eta_p^2 = 0.05$, 95% CI = (0.00, 0.15)).

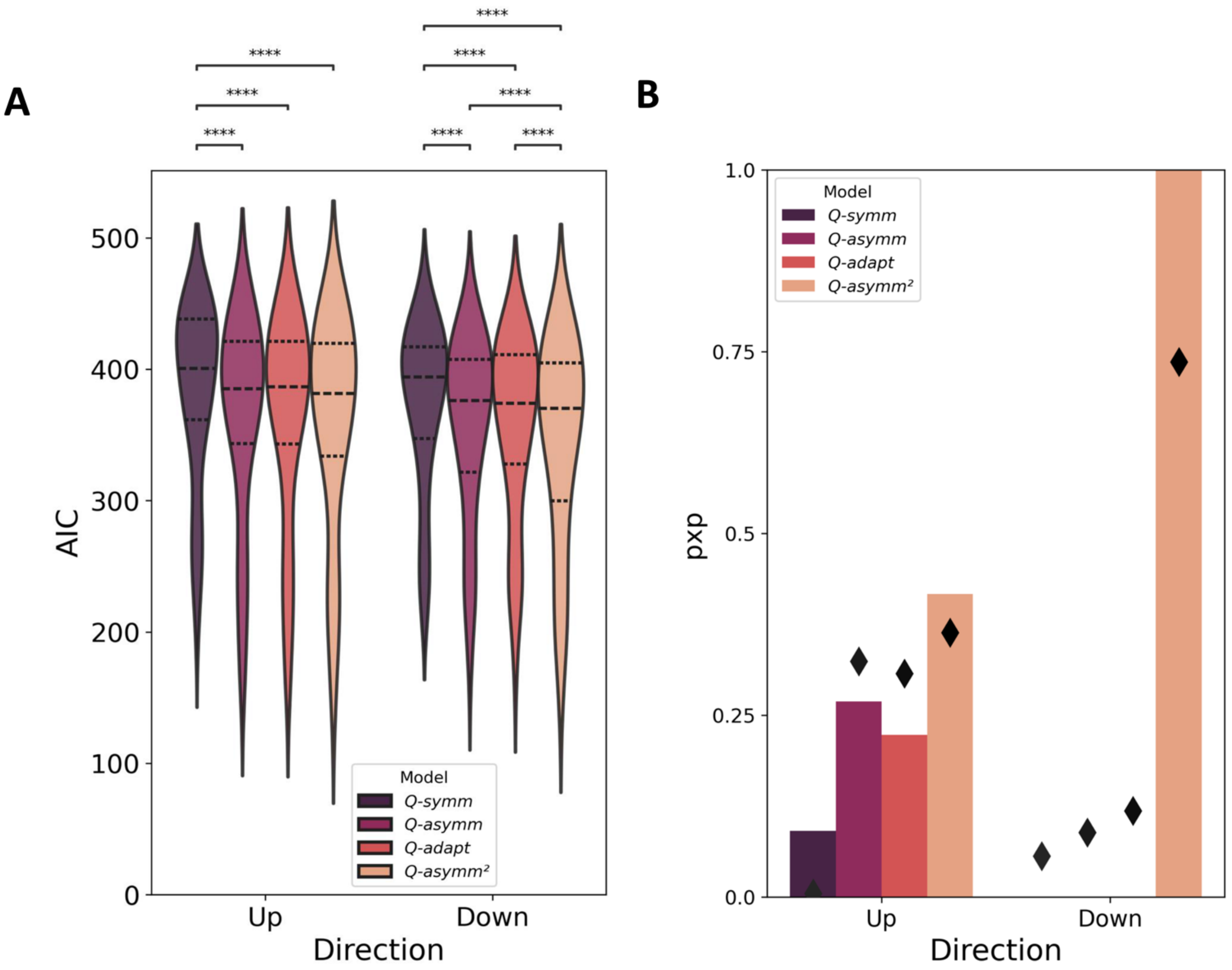

Fig S9A-B. Model comparison for each candidate model, within each task condition, for the larger participant set. Same as Fig 5A-B, but for the participant sample obtained after the application of a more liberal performance-related exclusion criterion (i.e. N = 103, rather than N = 83). As in the more conservative sample reported in the main text, *Q-asymm*^{2} provided the best fit to participant data ('up': *Q-asymm*^{2}: pxp = 0.42; *Q-adapt*: pxp = 0.22; *Q-asymm*: pxp = 0.27; *Q-symm*: pxp = 0.09; 'down': *Q-asymm*^{2}: pxp > 0.99; *Q-adapt*: pxp < 0.01; *Q-asymm*: pxp < 0.01; *Q-symm*: pxp < 0.01).

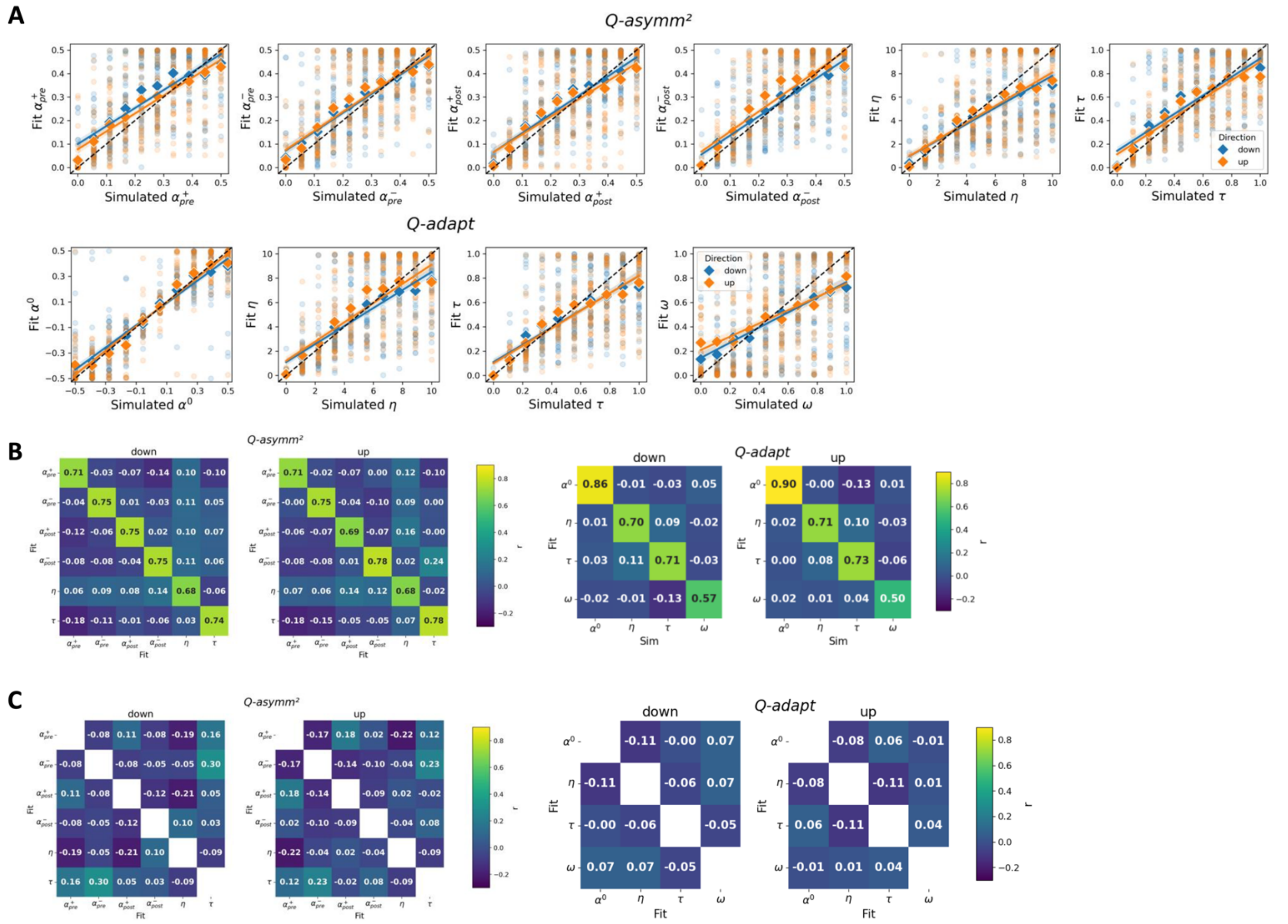

Fig S10A-B. **Parameter recovery analysis**. Our two winning models *Q-asymm*^{2} and *Q-adapt* exhibited strong parameter recovery. In other words, simulated parameters (columns) correlated strongly with their recovered counterparts (rows; **A** and diagonal in **B**), and correlated weakly with each other recovered parameter type (**B** off-diagonal)**.** In addition, we observed weak correlations *among* recovered parameters, as shown in **C**. See *Supplementary Methods – Model and Parameter Recovery* for details.

Supplementary Methods – Model and Parameter Recovery

An important prerequisite for comparing models fitted to empirical data is that they are identifiable – that is, such models should behave in ways that renders them distinguishable under the selected model evidence metric [2]. To validate our model comparison approach, we first took each model's best-fitting parameters for each of the 83 participants, and used these to generate 10 experimental runs of synthetic (binomial) choice data on each participant's set of trial sequences. We then fitted each model to the generated data and evaluated how often each model provided the best fit. The resulting confusion matrix thus provides a measure of the conditional probability that a model fits the data best, given the true generative model: *p(fit|gen)*. In turn, this allows one to 'invert' the confusion matrix according to Bayes rule, under the assumption of a uniform prior over all models:

$$p(gen|fit) = \frac{p(fit|gen)p(gen)}{\sum_{sim=1}^{nModels} p(fit|gen)_{sim} p(gen)_{sim}} \qquad \text{Eq. 11}$$

In quantifying the probability that the data were generated by a specific model, given that this model provided the best fit to the generated data, the inverted confusion matrix helpfully complements the model recovery procedure. As can be observed in the AIC-based confusion and inverted confusion matrices (Fig S4A-B), our models of interest exhibited reliable recoverability in both 'down' and 'up' task settings, albeit with moderate confusion of *Q-asymm* and *Q-adapt*. We note that model separability was greatly improved when using the AIC relative to when using (inverted) confusion matrices that used the Bayesian Information Criterion (BIC)[3], which over-penalised *Q-asymm* and *Q-asymm^2's* additional free parameter(s) (Fig S4C-D). Thus, we used AIC as our approximation of model evidence.

Finally, to validate inferences about empirical parameters obtained from our model fitting procedure, we simulated choice behaviour under our two key 'winning' models of interest, *Q-asymm^2* and *Q-adapt*, using the best-fitting parameter settings estimated for each participant, and then re-fit these models to these generated datasets [2]. We then repeated the process while incrementally varying each of the data-generating parameters over 10 evenly spaced values within the lower and upper bounds used for our model fitting procedure (see 'Model Fitting and Comparison'). In both task conditions and model types, the 'true', data-generating parameters strongly correlated with their recovered counterparts (min r = 0.50, max r = 0.90), and only weakly correlated with all other recovered parameter types (min r = -0.18, max r = 0.16; Fig S6A-B). Likewise, we observed only weak correlations among the recovered parameters themselves (min r = -0.22, max r = 0.30), indicating that our fitting procedure did not introduce any 'trading off' among parameters, and thus further validating inferences drawn about these parameters (Fig S10A-C)[2,4].

Supplementary Note 1: *Q-adapt*

Our analysis of *Q-asymm*'s fit to the behavioural data indicated that while this model provided a good overall fit to both groups of participants' behaviour, especially with respect to pre-changepoint trials, it was limited in its ability to account for well-performing participants who initially exhibited value compression, but who were nonetheless capable of responding appropriately to the downward change in relational structure. We therefore sought to explore how *Q-asymm* might be modified to make its learning policy flexible enough to capture the behaviour of such participants.

Inspiration for how differing degrees of asymmetry may arise as a function of some relevant task feature came from Ciranka et al.'s [1] finding that the sparsity of feedback appears to play a role in modulating learning policy asymmetry. Specifically, they observed that whereas participants tended to exhibit asymmetric belief-updating policies in the standard partial feedback TI paradigm, performance in a task offering full feedback on *all* comparisons, as opposed to just comparisons between neighbours, was best characterised by symmetric learning rates, and hence best fit by the symmetric model *Q-symm*. Such feedback regimes facilitate learning because they offer participants the opportunity to learn the cnarciness relations between non-neighbouring items directly. This also provides many more opportunities for the agent to confirm or revise their prior beliefs about the ordinal positions of the item set, which may lend itself to the application of symmetric updates to both compared items on a given trial. In contrast, in partial feedback settings where participants are required to 'build' a representation of the transitive hierarchy purely endogenously, the paucity of feedback that verifies or falsifies the agent's beliefs about the ranking of items may necessitate asymmetrically prioritising the update of just one of the two compared items on a given comparison until a clearer representation of the item hierarchy has been formed.

We therefore formalised an adaptive agent *Q-adapt*, whose degree of asymmetry varied on a trial-by-trial basis as a function of the strength or uncertainty of the agent's preference regarding the cnarciness relation between the two compared items. The rationale was that trials for which the agent's preference for one of the two items is less certain may induce them to (asymmetrically) allocate a larger proportion of the overall update to one of the items. In contrast, on trials where the agent has a stronger preference, the receipt of feedback should provide a clear indication that this prior belief needs to be further reinforced or reversed via a more symmetrically distributed updating of both items. Drawing on the information theoretic notion of choice entropy, we derived an asymmetry variable λ which is a function of the absolute strength of choice preference for one of two items in the current pair, and controls the degree to which the agent's 'base' learning rate resource α^0 is shared between α^+ and α^- (Fig S5A; see Eqs. 6-8 in *Materials and Methods,* 'Behavioural Models'). For example, assuming an agent with a general tendency towards winner-biased updates, when λ is 1 (corresponding to a weak choice preference), all of α^0 will be allocated to α^+, whereas α^- is set to 0. As λ approaches 0 (corresponding to a stronger choice preference), however, α^0 is more evenly spread across both learning rates, meaning α^+ and α^- become more symmetrical. Thus, whereas *Q-asymm* defines α^+ and α^- as two free parameters, *Q-adapt* has a single base learning rate parameter α^0 that is adaptively spread between α^+ and α^- as a function of λ on a trial-by-trial basis.

In dynamically distributing learning updates in this way, *Q-adapt* models participants as tending to be more asymmetric in their updates towards the beginning of the experiment while they are still learning the transitive hierarchy, thus mirroring *Q-asymm's* asymmetric policy. As learning progresses, and hence stronger (and, ideally, correct) beliefs about item relations are formed, learning updates are distributed more symmetrically (note that as the agent's expectations about item relations become more accurate, this will in turn reduce the relative difference between predicted item values, resulting in a concomitant reduction in learning, as per Eq. 4). By the

time the changepoint is reached and the agent observes that $i_7<i_1$ – i.e. an outcome that contradicts the agent's strong prior belief that $i_1<i_7$ –, the symmetric nature of the quadratic function allows for an updating of both $Q(i_1)$ and $Q(i_7)$ that is itself more symmetric, albeit still winner-biased. This is consistent with our finding that participants of both groups were equally capable of repositioning the moved anchor in each case, despite the differential impact of the changepoint on downstream TI performance.

The extent to which an agent may tend towards such symmetric updates is not only shaped by an additional sensitivity parameter ω (see Eq. 6), but also depends on how readily the agent forms strong preferences. This is itself determined by several interacting factors, including the rate at which the agent updates items upon receipt of new feedback (i.e. the learning rate), and the behavioural variability arising from the decision process (i.e. the temperature parameter τ of the logistic choice function; see Eq. 5 in *Materials and Methods,* 'Behavioural Models'). In the present case, well-performing agents, such as those with lower values of τ will tend to more readily translate differences in value estimates into stronger choice preferences (Fig S5B), and hence will be more inclined to distribute more symmetric updates as learning progresses via lower values of λ (Fig S5C). In contrast, noisier agents will tend towards more asymmetric updates, limiting their ability to adapt to the change in relational structure occurring in the 'down' group. Thus, in modulating learning asymmetry as a function of choice preference, which is itself shaped by internal learning and noise parameters, the present implementation of *Q-adapt* allows agents to a) initially exhibit asymmetric learning while choice preferences are being acquired, and (crucially), b) appropriately deploy more symmetric learning later on in the learning phase under 'well-performing' learning and choice parameterisations (e.g. via a low decision noise parameter).

We also considered an alternative version to *Q-adapt* in which the asymmetry modulator λ is simply an entropy function of the choice preference strength, such that Eq. 6 in *Methods*, 'Behavioural Models' is replaced by the following:

$$\lambda_t = -p_t(x > y)\log_2(p_t(x > y)) - -(1 - p_t(x > y)\log_2(1 - p_t(x > y)) \quad \text{Eq. 9}$$

However, fitting this model to participant data revealed a significantly worse fit relative to the original 'quadratic' variant of *Q-adapt* described in Eq. 6 ('quadratic' *Q-adapt*: mean AIC = 344.06 ± 8.14 SE; 'entropy' *Q-adapt*: mean AIC = 348.11 ± 7.71 SE; Wilcoxon signed-rank test of AICs: z = 2.17, p = .030, r = 0.24, 95% CI = (0.03, 0.43). Given this inferior predictive performance for the 'entropy' model variant of *Q-adapt*, we excluded it from our formal model comparison.

Supplementary References

1. Ciranka S, Linde-Domingo J, Padezhki I, Wicharz C, Wu CM, Spitzer B. Asymmetric reinforcement learning facilitates human inference of transitive relations. Nat Hum Behav. 2022 Apr;6(4):555–64.

2. Wilson RC, Collins AG. Ten simple rules for the computational modeling of behavioral data. eLife. 2019 Nov 26;8:e49547.

3. Schwarz G. Estimating the Dimension of a Model. The Annals of Statistics. 1978 Mar;6(2):461–4.

4. Daw ND. Trial-by-trial data analysis using computational models. In: Decision Making, Affect, and Learning: Attention and Performance XXIII. Oxford University Press; 2011. p. 3–38.
