# Supplementary material for: Asymmetric learning and adaptability to changes in relational structure during transitive inference
Source: Commun Psychol. 2025 Nov 14;3:155. doi: 10.1038/s44271-025-00352-0 (PMC12618241; doi:10.1038/s44271-025-00352-0)
Supplement: Supplementary file 3 — Reporting summary [file 44271_2025_352_MOESM3_ESM.pdf]

nature portfolio

Corresponding author(s): Thomas Graham

Last updated by author(s): Sep 16, 2025

Reporting Summary

Nature Portfolio wishes to improve the reproducibility of the work that we publish. This form provides structure for consistency and transparency in reporting. For further information on Nature Portfolio policies, see our Editorial Policies and the Editorial Policy Checklist.

Statistics

For all statistical analyses, confirm that the following items are present in the figure legend, table legend, main text, or Methods section.

n/a	Confirmed	
☐	☒	The exact sample size (*n*) for each experimental group/condition, given as a discrete number and unit of measurement
☐	☒	A statement on whether measurements were taken from distinct samples or whether the same sample was measured repeatedly
☐	☒	The statistical test(s) used AND whether they are one- or two-sided<br>*Only common tests should be described solely by name; describe more complex techniques in the Methods section.*
☒	☐	A description of all covariates tested
☐	☒	A description of any assumptions or corrections, such as tests of normality and adjustment for multiple comparisons
☐	☒	A full description of the statistical parameters including central tendency (e.g. means) or other basic estimates (e.g. regression coefficient) AND variation (e.g. standard deviation) or associated estimates of uncertainty (e.g. confidence intervals)
☐	☒	For null hypothesis testing, the test statistic (e.g. *F*, *t*, *r*) with confidence intervals, effect sizes, degrees of freedom and *P* value noted<br>*Give P values as exact values whenever suitable.*
☒	☐	For Bayesian analysis, information on the choice of priors and Markov chain Monte Carlo settings
☒	☐	For hierarchical and complex designs, identification of the appropriate level for tests and full reporting of outcomes
☐	☒	Estimates of effect sizes (e.g. Cohen's *d*, Pearson's *r*), indicating how they were calculated

Our web collection on statistics for biologists contains articles on many of the points above.

Software and code

Policy information about availability of computer code

Data collection	Psychopy (https://www.psychopy.org/builder/builder.html); custom code: https://github.com/tgham/asymm_switch
Data analysis	Python (https://www.python.org/); custom code: https://github.com/tgham/asymm_switch

For manuscripts utilizing custom algorithms or software that are central to the research but not yet described in published literature, software must be made available to editors and reviewers. We strongly encourage code deposition in a community repository (e.g. GitHub). See the Nature Portfolio guidelines for submitting code & software for further information.

Data

Policy information about availability of data

All manuscripts must include a data availability statement. This statement should provide the following information, where applicable:

- Accession codes, unique identifiers, or web links for publicly available datasets
- A description of any restrictions on data availability
- For clinical datasets or third party data, please ensure that the statement adheres to our policy

The data that support the findings of this study are available at dx.doi.org/10.6084/m9.figshare.26147470

Human research participants

Policy information about studies involving human research participants and Sex and Gender in Research.

Reporting on sex and gender	No sex- or gender-based analysis was conducted
Population characteristics	Participants (N=150) aged between 18-40 years were recruited online via Prolific Academic (74 female; mean age 27 ± 5.27 years SE). After the application of exclusion criteria (see Materials and Methods), N=83 participants (36 female; mean age = 26.90 ± 5.34 years SE) remained for analysis ('up': N=39; 'down': N=44).
Recruitment	Participants were recruited online via Prolific Academic (www.prolific.co). After confirming their written informed consent, participants were randomly allocated to one of two groups: the ‘up’ group (N=76; 37 female; mean age = 27.14 ± 5.12 years SE), or the ‘down’ group (N=74; 37 female; mean age = 26.85 ± 5.41 years SE). Participants received compensation of £6.00, plus a performance-dependent bonus of £2.
Ethics oversight	All experiments were approved by the ethics committee of the Max Planck Institute for Human Development.

Note that full information on the approval of the study protocol must also be provided in the manuscript.

Field-specific reporting

Please select the one below that is the best fit for your research. If you are not sure, read the appropriate sections before making your selection.

☐ Life sciences ☒ Behavioural & social sciences ☐ Ecological, evolutionary & environmental sciences

For a reference copy of the document with all sections, see nature.com/documents/nr-reporting-summary-flat.pdf

Behavioural & social sciences study design

All studies must disclose on these points even when the disclosure is negative.

Study description	The study consists of one online behavioural experiment that used a between-subjects design (two groups: 'up' and 'down'). Computational models were simulated and fit to the collected behavioural data.
Research sample	Participants (N=150) aged between 18-40 years were recruited online via Prolific Academic (74 female; mean age 27 ± 5.27 years SE). Participants were fluent in English. After confirming their written informed consent, participants were randomly allocated to one of two groups: the ‘up’ group (N=76; 37 female; mean age = 27.14 ± 5.12 years SE), or the ‘down’ group (N=74; 37 female; mean age = 26.85 ± 5.41 years SE). We used the Prolific setting for collecting representative samples, such that the study was distributed based on UK/US census data.
Sampling strategy	Participants were recruited by opportunity sampling, and were randomly assigned to each experimental condition. The sample size was based on sample sizes of a previously published study that collected online behavioural data to examine belief-updating asymmetries during relational learning.
Data collection	The experiment was distributed online using Prolific. Qualtrics was used to record basic demographics and informed consent from participants.
Timing	100 participants 25th-26th October 2023; 50 participants 1st-2nd February 2024
Data exclusions	We used a binomial test to compute a performance threshold above which the likelihood that participants were performing at chance on pre-changepoint trials was 0.01 (i.e. following the criteria used by a similar study), thus avoiding a confound by the experimental manipulation of interest. One additional participant was excluded for exhibiting a high proportion of missed responses (>60% of 322 trials). After the application of these criteria, N=83 participants (36 female; mean age = 26.90 ± 5.34 years SE) remained for analysis ('up': N=39; 'down': N=44). Restricting the application of this criterion to the first half of the experiment while participants were still learning to perform the task amounted to a somewhat conservative approach, in turn resulting in a relatively high proportion of participants being excluded. Nonetheless, we note that when we applied a more liberal threshold for inclusion (a = 0.1), which left N=103 participants ('up': N=53; 'down': N=50), our core findings remained unchanged.
Non-participation	11 participants signed up for the study online but did not complete the experiment.
Randomization	Participants were randomly assigned to each of the between-subjects conditions. Stimuli were randomly assigned a ground truth rank for each participant. The serial order of trials was pseudo-randomised, with left and right positions counterbalanced within each block.

Reporting for specific materials, systems and methods

We require information from authors about some types of materials, experimental systems and methods used in many studies. Here, indicate whether each material, system or method listed is relevant to your study. If you are not sure if a list item applies to your research, read the appropriate section before selecting a response.

Materials & experimental systems

n/a	Involved in the study
☒	☐ Antibodies
☒	☐ Eukaryotic cell lines
☒	☐ Palaeontology and archaeology
☒	☐ Animals and other organisms
☒	☐ Clinical data
☒	☐ Dual use research of concern

Methods

n/a	Involved in the study
☒	☐ ChIP-seq
☒	☐ Flow cytometry
☒	☐ MRI-based neuroimaging
